# Atrial Fibrillation in Scrub Typhus: A Series of Four Cases

**DOI:** 10.7759/cureus.25338

**Published:** 2022-05-25

**Authors:** Harshit Gupta, Ashwin Parchani, Arnab Choudhury, Jithesh G, Monika Pathania, Mukesh Bairwa

**Affiliations:** 1 Department of Internal Medicine, All India Institute of Medical Sciences, Rishikesh, IND

**Keywords:** ischemic heart disease, myocarditis, arrhythmia, atrial fibrillation, scrub typhus

## Abstract

Scrub typhus, also known as bush typhus, is an acute febrile zoonosis caused by *Orientia tsutsugamushi*, transmitted by the bite of chigger mite. Patients with scrub typhus can have many different presentations such as acute hearing loss, interstitial pneumonitis, acute respiratory distress syndrome, myocarditis, pericarditis, meningoencephalitis, acute renal failure, acute hepatic failure, and septic shock. The occurrence of multi-organ dysfunction is responsible for high mortality seen in scrub typhus patients. Cardiovascular involvement can also occur in the form of arrhythmia, which leads to an increase in mortality in these patients, and if associated with ischemic heart disease and acute heart failure, it leads to higher mortality. The early use of antibiotics and telemetry monitoring along with aggressive management of patients can decrease the complications and mortality seen in these patients. This study describes a series of four scrub typhus patients with new-onset atrial fibrillation who were managed with either direct current (DC) cardioversion, amiodarone, or diltiazem.

## Introduction

*Orientia tsutsugamushi*, an obligate intracellular Gram-negative bacterium, is known to cause life-threatening zoonosis scrub typhus this disease is transmitted to human beings by the bite of infected larval trombiculid mite of genus Leptotrombidium [1]. it is endemic to Asia, especially in regions such as India, Pakistan, Japan, Taiwan, Korea, China, Thailand, Malaysia, and tropical parts of Australia [2,3]. it is known to involve multiple systems leading to acute kidney injury, meningitis, encephalitis, relative bradycardia, pericardial effusion, and superficial ulcers in the gastrointestinal tract [4-8]. The patient usually presents with fever, eschar with black crust, headache, myalgia, cough, and lymphadenopathy after an incubation period of seven to 10 days [9].

There have been reports of the cardiovascular system involvement in scrub typhus leading to relative bradycardia [6], pericardial effusion [7], cardiomegaly [10], ischemic heart disease [11], and ST changes [12]. Some reports have even outlined new-onset ECG changes suggestive of atrial fibrillation (AF), torsades de pointes, and atrial flutter in these patients [13]. According to a study done in Thailand, there was nine new-onset atrial fibrillation among 79 scrub typhus patients while in another study there were 13 cases of atrial fibrillation reported from a total of 165 scrub typhus patients [13,14]. Here, the authors presented four cases of scrub typhus presenting with atrial fibrillation with their respective clinical profiles.

## Case presentation

Case 1

**Figure 1:**
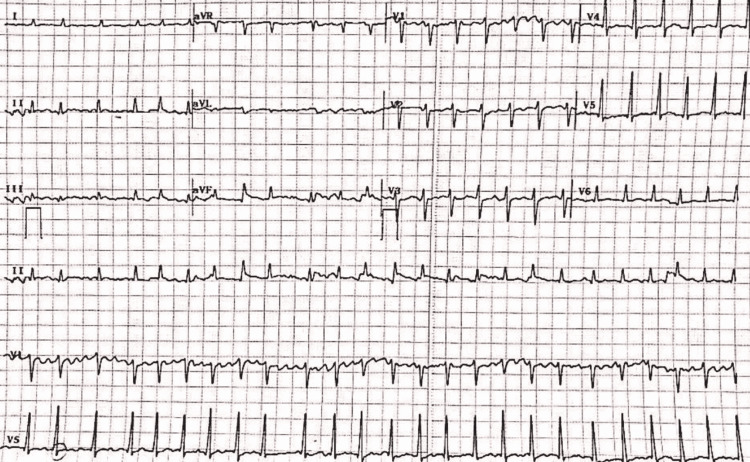
The image shows irregularly irregular rhythm suggestive of atrial fibrillation.

A 69-year-old male chronic smoker for 40 years and occasional alcohol use, presented to the emergency department with a seven-day history of acute onset, intermittent, moderate grade fever associated with chills and rigor, which was relieved with medication. This was associated with acute productive cough with hemoptysis, yellowish discoloration of the sclera, and altered sensorium for three days. On examination, the patient was febrile and normotensive with associated tachycardia. Bilateral pitting non-tender pedal edema was noted. The patient was evaluated for acute febrile illness. Labs were notable for transaminitis with a cholestatic pattern of liver involvement and deranged renal function (Table 1). Workup for tropical infections also came out positive for scrub typhus. The patient was initiated on doxycycline, but the condition worsened further, and he developed an irregularly irregular rhythm on electrocardiography suggestive of atrial fibrillation (Figure 1). The patient was treated with intravenous amiodarone, and rhythm control was achieved. During the hospital stay, he developed worsening sensorium and multi-organ dysfunction and was shifted to ICU for the need for mechanical ventilation and eventually succumbed to the illness.

**Table 1:** Results of complete blood count, liver function test, and renal function test.

Investigations	Reference range, adults (this hospital)	Patient 1	Patient 2	Patient 3	Patient 4
Hemoglobin (g/dL)	13.5-17.5	9.69	13.8	15.35	14.3
White cell count (per μL)	4500-11000	5875	20480	5011	13760
Differential count (per μL)					
Neutrophils	40-80%	44	83.12	55	87
Lymphocytes	20-40%	50	9.45	30	8
Monocytes	2-10%	4.91	6.42	9	1
Platelets (per μL)	150000-400000	18000	86000	70000	110000
Liver function tests					
Aspartate aminotransferase (U/L)	5-40	223	127	219	129.4
Alanine aminotransferase (U/L)	5-45	105	140	123	137.8
Total bilirubin (mg/dL)	0.2-1.1	2.72	2.1	2.43	3.17
Direct bilirubin (mg/dL)	<0.20	1.77	1.35	1.67	2.13
Alkaline phosphatase (U/L)	<240	291	210	677	1483
Gamma-glutamyl transferase (U/L)	0-55 U/L	70	172	102	466
Renal function test					
Sodium (mmol/L)	135-145	149	135	132	137
Potassium (mmol/L)	3.5-5.0	2.6	4.2	5.2	6.5
Blood urea (mg/dL)	17-43	110	249	120	227
Creatinine (mg/dL)	0.72-1.18	1.46	5.68	1.35	4.75

Case 2

**Figure 2:**
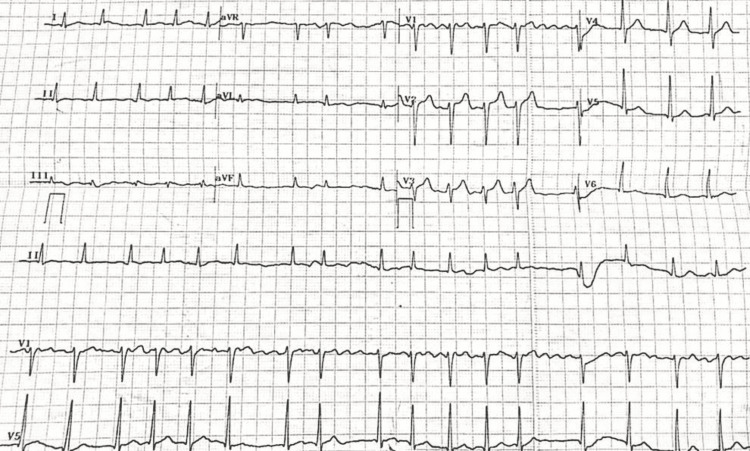
ECG showing absent p waves and irregularly irregular rhythm suggestive of atrial fibrillation.

A 56-year-old male, an occasional alcoholic, was asymptomatic 15 days ago when he experienced a sudden onset of fever with chills and rigor, with no discernible pattern. This was associated with acute onset persistent headache that lasted for three days and was relieved on its own. There was insidious onset, progressive, diffuse, dull aching pain in the abdomen, which was non-radiating, increased on lying down, and associated with acute onset vomiting after food intake, which was non-bloody, non-bilious. He had yellowish discoloration of urine and altered mental sensorium in the form of irrelevant self-talking without any loss of consciousness, abnormal body movement, focal neurological deficit, or meningeal signs. He had an acute onset progressive non-exertional shortness of breath associated with orthopnea. There was no associated cough, chest pain, palpitations, and pedal edema. It was associated with acute onset decreased urine output, progressive, painless, and without hematuria, frothuria, dysuria, urine urgency, and urine frequency. On examination, eschar was noted below the umbilicus in the lower abdomen, and wheezes were present. Initial investigation revealed thrombocytopenia, deranged renal function, acute liver injury, and IgM scrub came out to be positive (Table 1). The patient's conditions worsened during the hospital stay, and he developed atrial fibrillation suggested by irregularly irregular rhythm on ECG (Figure 2). The patient was administered PO diltiazem and rate control was achieved.

Case 3

**Figure 3:**
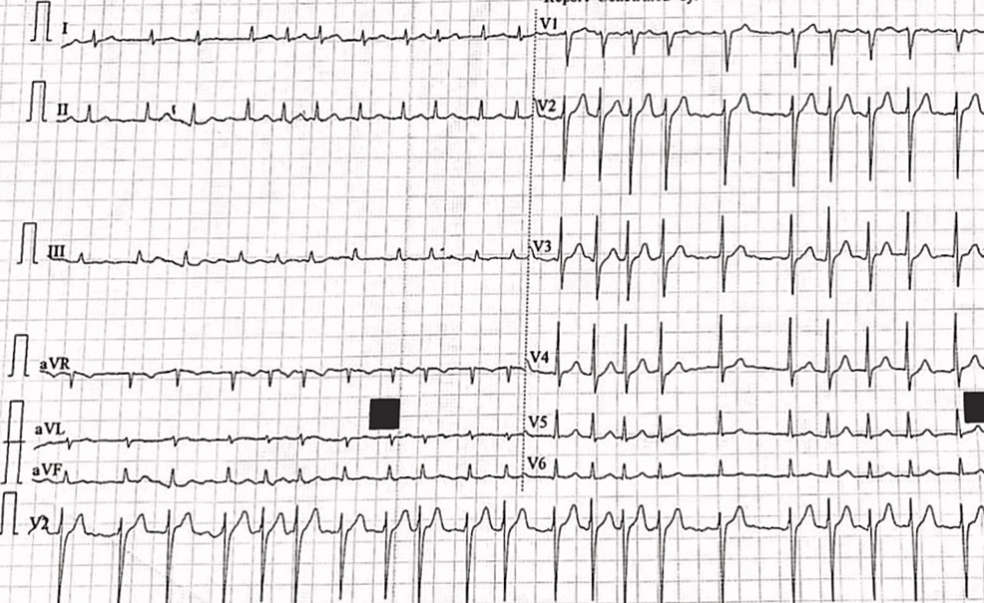
ECG showing irregularly irregular rhythm suggestive of atrial fibrillation.

A 50-year-old male with a past history of tuberculosis presented with acute onset, high grade documented (103°F) fever for 10 days, associated with chills and rigor, no diurnal variation was present, and was not relieved on taking medication. It was associated with orthopnea, paroxysmal nocturnal dyspnea (PND), and palpitation. It was not associated with cough. On examination, cyanosis was present and coarse crepitus was present. Initial investigation revealed thrombocytopenia, renal function deranged, acute liver injury and IgM scrub came out positive (Table 1). The patient was started on non-invasive ventilation. He developed sudden onset, non-radiating retrosternal chest pain, which was associated with palpitation and diaphoresis. ECG revealed irregularly irregular rhythm suggestive of atrial fibrillation (Figure 3). Direct current (DC) cardioversion (120 Joules) was performed under ketamine sedation. The patient was conservatively managed after the rhythm resolved. The patient's oxygen requirement decreased, and non-invasive ventilation was tapered off. Two-dimensional echocardiography (2D Echo) showed normal ejection fraction with no left ventricular regional motion wall abnormality. Creatine kinase-MB (CK-MB) was also found to be increased (38 IU/L).

Case 4

**Figure 4:**
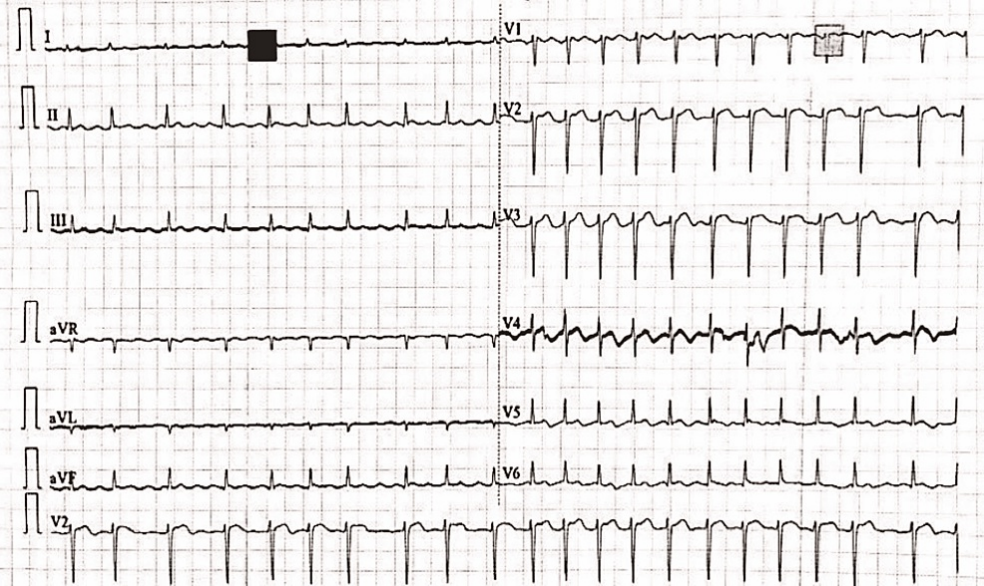
ECG showing irregularly irregular rhythm suggestive of atrial fibrillation.

A 71-year-old female reformed smoker, known case of hypertension for 1.5 years (not on any medication) presented with acute onset, documented (102°F) fever for 12 days, which was associated with hyperactive delirium and melena, and was intubated in the emergency department due to low Glasgow Coma Scale (GCS). In the emergency room, the patient experienced an episode of right-side focal seizure. She was then transferred to the intensive care unit. Investigations revealed impaired renal function, acute liver injury, and a positive IgM scrub (Table 1). An upper gastrointestinal endoscopy revealed duodenal ulcers. Cerebrospinal fluid (CSF) analysis revealed protein levels of 110 mg/dL, sugar levels of 120 mg/dL, and a total leukocyte count of 80 cells/μL (90% monomorphic). The patient was started on doxycycline after being diagnosed with scrub typhus. During her hospital stay, her condition worsened and she developed an irregularly irregular pulse. An ECG was performed, which revealed atrial fibrillation (Figure 4). The patient was given DC cardioversion with 120 Joules in view of compromised hemodynamic status. The abnormal rhythm resolved and the patient was managed conservatively afterward.

## Discussion

Scrub typhus is known to involve the cardiovascular system leading to a pericardial effusion [7], myocarditis [15], myocardial infarction [16], and findings in ECG like ischemic changes, arrhythmia, and QT prolongation were found in the patients with scrub typhus [13].

In this study, the patients presented with acute onset fever, and they were evaluated in terms of acute febrile illness. Labs suggested acute renal injury and acute liver injury. IgM scrub came out to be positive. The patients were started on doxycycline therapy. During their hospital stay, the patients’ condition deteriorated, and they developed abnormal rhythm; when ECG was done, it revealed changes suggestive of atrial fibrillation. Two of the four patients underwent cardioversion because of hemodynamic instability, while the other two were medically managed with amiodarone and diltiazem. One mortality was reported among the four patients due to multi-organ dysfunction. In one of the four patients, hypokalemia was present, which may be an independent risk factor for atrial fibrillation, necessitating laboratory assessment for serum electrolytes disturbances.

A cohort study previously conducted in scrub typhus patients revealed new-onset atrial fibrillation in 1% of the scrub typhus patients. Out of this 1% of patients who had new-onset atrial fibrillation, 87.2% of patients were aged more than 65 years [11]. This study also reported a 1.3-fold increase in three months mortality in new-onset atrial fibrillation patients, and this mortality further increased when associated with complications like acute heart failure (2.4 fold) and ischemic heart disease (13.7 fold) [11]. The studies previously conducted in scrub typhus patients have also reported ECG alterations like ST-segment elevation, AV block, PR-segment depression, and T wave inversions [17].

Scrub typhus post-inoculation from bite spreads to regional lymph nodes and leads to lymphadenopathy. The involvement of blood vessels leads to vasculitis, which may further lead to target organ damage. Similarly, it leads to inflammatory changes in the myocardium, leading to structural and functional damage responsible for the ECG changes like atrial fibrillation seen in scrub typhus patients [11]. Cardiovascular involvement with myocarditis and ECG changes has also been reported in other topical infections like dengue, with pathomechanisms attributed to macrophage activation, immune-mediated cardiomyocyte damage, direct invasion of cardiomyocytes, and electrolyte alterations [18].

In scrub typhus patients’ the mainstay of treatment is antibiotics. Doxycycline is the antibiotic of choice, and in resistant cases of scrub typhus rifampicin and azithromycin are used. Early antibiotic use leads to a decrease in mortality (from 6% to 1.4%) and complications [12]. In scrub typhus patient who develops atrial fibrillation, along with doxycycline cardioversion is done in hemodynamically unstable patients, while in hemodynamically stable patients rate-controlling agents like calcium channel blockers, beta-blockers, and if rate control does not occur or the patient is symptomatic, rhythm control with cardioversion or antiarrhythmic like amiodarone, flecainide, propafenone, dofetilide, and intravenous ibutilide are used [19].

Our study observed that atrial fibrillation can be seen in scrub typhus patients, therefore emphasizing the importance of serial ECG monitoring in such patients to look for the presence of any abnormalities. Early detection and management of the presence of any such arrhythmias is vital and can influence the prognosis of the condition.

## Conclusions

In conclusion, scrub typhus infection can cause inflammation of the myocardium, precipitating new-onset atrial fibrillation with rapid ventricular response, leading to an increase in mortality and complications in these patients. Such patients require telemetry monitoring to monitor rhythm and aggressive treatment with either rate control or rhythm control agents depending upon the patient's condition. In addition, early use of antibiotics can decrease the mortality and complications in these patients.
